# Public health and integrated governance across the sustainable development goals beyond 2030: a six-function framework

**DOI:** 10.3389/fpubh.2026.1892248

**Published:** 2026-07-17

**Authors:** Nurah Alamro

**Affiliations:** 1 Department of Family and Community Medicine, College of Medicine, King Saud University, Riyadh, Saudi Arabia; 2 Department of Family and Community Medicine, King Saud University Medical City, King Saud University, Riyadh, Saudi Arabia

**Keywords:** health equity, health in all policies, implementation science, integrated governance, intersectoral action, policy coherence, public health policy, sustainable development goals

## Abstract

The Sustainable Development Goals (SDGs) define sustainable development as integrated and indivisible, yet implementation remains organized largely through sectoral mandates, budgets, indicators, and accountability systems. This fragmentation is especially consequential for public health because progress on SDG 3 (Good Health and Wellbeing) depends on policy choices across social, economic, environmental, and fiscal domains. The institutional capacities required to act on these interdependencies remain insufficiently specified. This review synthesizes evidence from SDG-interlinkage research, policy coherence for sustainable development, public health governance, health-system strengthening, implementation science, and governance theory. It compares three pathways for the period beyond 2030: enhanced interlinkage reporting; stronger policy-coherence and multisectoral mechanisms; and integrated governance supported by explicit institutional functions. Integrated governance for sustainable development denotes arrangements that bring cross-sector effects into decision-making, financing, measurement, accountability, and learning. The proposed framework comprises six functions: coherence, arbitration, shared-outcome financing, interlinkage-aware measurement, equity-sensitive accountability, and adaptive learning. The analysis focuses on public health because health outcomes and inequities reflect the cumulative effects of decisions across sectors; the framework may also inform other policy domains characterized by similar interdependence. The review specifies the institutional capacities needed to govern interdependencies already recognized within the SDG framework.

## 1 Introduction

The Sustainable Development Goals (SDGs) were conceived as an integrated and indivisible agenda (1). Their delivery remains embedded in institutions organized by sector, each with its own mandate, budget, indicators, and lines of accountability. This architecture obscures the pathways through which progress in one domain can impose costs elsewhere and allows risks to accumulate across populations and over time. The Global Sustainable Development Report 2023 judged incremental and fragmented action inadequate to the transformations required, and the Pact for the Future renewed the call for action across government and society (2, 3). The approach of 2030 brings an established institutional problem into sharper focus: whether the next development framework will change how interdependence is governed.

Public health offers a particularly informative vantage point. SDG 3 is the Sustainable Development Goal concerned with Good Health and Wellbeing. Progress toward this goal depends on decisions across environmental, social, economic, and fiscal policy (4). Health outcomes absorb the cumulative consequences of those decisions; health inequities show how exposure and protection are distributed. Public health therefore reveals whether development institutions can recognize cross-sector effects and respond before they translate into preventable harm and widening inequity.

A substantial literature has mapped interactions among the SDGs and documented synergies, trade-offs, spillovers, and variation across contexts (5–18). The remaining gap concerns institutional action. Evidence of interdependence must enter policy appraisal, inform the exercise of authority when objectives conflict, shape the allocation of resources, and establish responsibility for effects that cross sectoral boundaries. It must also alter implementation when policies produce unintended harm. Interlinkage analysis alone does not provide these capacities.

Public health has addressed versions of this problem for decades through intersectoral action, Health in All Policies, and multisectoral governance (19–24). Their experience shows that convening institutions has limited effect when mandates and incentives remain separate. Cross-sector action also weakens when financing and accountability remain attached to individual agencies rather than shared outcomes. These lessons provide a foundation for examining the governance of interdependence across sustainable development more broadly.

This review uses integrated governance for sustainable development to describe the institutional arrangements through which sustainable-development objectives are pursued when policy effects extend across sectors, populations, places, and time horizons. It compares three options for the period beyond 2030 and develops a framework comprising coherence, arbitration, shared-outcome financing, interlinkage-aware measurement, equity-sensitive accountability, and adaptive learning. The framework specifies the institutional work required to make recognized interdependence consequential for policy and implementation. Although developed through public health, its relevance to other interdependent policy domains remains an empirical question.

## 2 Approach and evidence base

This review examines how institutions can translate interdependence across the Sustainable Development Goals into policy practice, drawing principally on public health. The synthesis was structured around four questions: how interactions among goals are understood; how governments align policy across sectors, levels of governance, borders, and time horizons; how public health traditions have approached intersectoral action; and how implementation systems adapt under conditions of complexity.

SDG interlinkage research shows that goals and targets interact through synergies, trade-offs, spillovers, and context-dependent effects (5–11). SDG governance scholarship identifies the collective-action, accountability, goal-setting, and implementation challenges created by global goals (12–14). Policy coherence for sustainable development provides concepts and mechanisms for aligning policy across sectors, levels of governance, borders, and time horizons (15–18). Public health governance traditions, including Health in All Policies, One Health, and multisectoral action, offer models for intersectoral decision-making, equity protection, and shared accountability (19–28). Systems thinking, health-system strengthening, and implementation science add complementary insights on complexity, resilience, feedback, adaptation, and the translation of evidence into practice (29–33). Governance theory explains why complex collective-action problems often require collaborative, polycentric, and institutionally embedded arrangements rather than coordination alone (34–36).

Across these evidence streams, we identified recurrent institutional tasks associated with acting on interdependence: aligning objectives, resolving trade-offs, allocating resources across sectoral boundaries, measuring cross-domain effects, accounting for distributional consequences, and adapting implementation in response to evidence. These tasks were consolidated into six governance functions and examined for their implications for public health and health equity (15–18, 21–24, 29–36).

The framework is a synthesis for policy analysis and institutional design rather than a validated measurement instrument or a claim that its component concepts are individually new. Equity is treated as a condition of credible integrated governance because cross-sector arrangements can reproduce existing power asymmetries when participation, transparency, and community knowledge remain weak or symbolic. In public health, the consequences of such failures are often borne by populations with the least authority to shape the decisions that affect them (18, 24, 27, 37).

## 3 The policy problem: integrated goals, fragmented institutions

The SDGs did not create interdependence; they made it explicit within a universal development framework. The 2030 Agenda established the integrated and indivisible character of sustainable development as a shared commitment, yet implementation has remained organized predominantly through sectoral institutions. Ministries retain separate mandates, budgets are negotiated through administrative silos, accountability systems reward sector-specific outputs, and SDG reporting commonly presents progress goal by goal. These arrangements can record progress while leaving unchanged the incentives that generate trade-offs, spillovers, and unequal exposure to harm (1, 5, 12–14).

This fragmentation is particularly consequential for public health because the determinants of health are distributed across policy domains. Climate policy shapes heat exposure, air quality, food security, infectious-disease risk, and health-system resilience (38). Urban and transport policy influence air pollution, injuries, physical activity, access to services, and social inclusion (39). Food systems affect nutrition, non-communicable disease risk, livelihoods, affordability, land use, and environmental sustainability (40). Social protection and labor policy influence poverty, food security, health service use, child development, and resilience to shocks (41). Public finance determines whether prevention, public health infrastructure, and universal health coverage can be sustained (30, 37).

Fragmented governance also creates externalities that may remain invisible within sectoral reporting. A policy can appear successful within one domain while transferring costs to another sector, population, territory, or generation. For public health, these costs may emerge as delayed disease burdens, inequitable exposure to environmental risks, fiscal pressure on health systems, or loss of trust among communities that bear the consequences of development choices without sharing in their benefits (14, 16–18, 37).

The policy problem lies in limited institutional capacity. Although the global agenda acknowledges that the goals interact, the arrangements through which those interactions influence policy appraisal, decision authority, financing, measurement, accountability, and implementation remain underdeveloped. An agenda beyond 2030 that improves reporting while leaving these institutional conditions unchanged risks documenting interdependence without governing it (12–18).

## 4 From sustainable development to integrated governance

### 4.1 Sustainable development remains the normative goal

Sustainable development remains the appropriate normative frame for the development agenda beyond 2030 because it connects human wellbeing, equity, dignity, and planetary conditions across generations (1, 37, 42, 43). Planetary health scholarship has strengthened this argument by showing how degradation of natural systems threatens human health and by calling for governance that links social, economic, and environmental policy domains (42). The Earth Commission has similarly emphasized the interdependence of planetary safety and social justice (43). From a public health perspective, the social determinants of health literature reinforces the same principle: avoidable differences in living conditions, power, and resources shape population health and health inequity (37).

This framing has direct implications for governance beyond 2030. A society may remain within aggregate environmental limits while distributing risks and protections unjustly. Conversely, policies that improve welfare in the short term can create delayed health, ecological, or fiscal burdens when planetary constraints are ignored (42, 43). Public health governance therefore requires institutions able to connect planetary boundaries, social distribution, and health equity within the same decision-making architecture (37, 42, 43).

The central issue is therefore how sustainable development can be delivered under conditions of complexity. The SDGs established a shared direction; the agenda beyond 2030 must strengthen the institutional capacity to act on interdependence, manage trade-offs, and protect equity across policy domains (1–3, 10–18).

### 4.2 Integrated governance for sustainable development

Integrated governance for sustainable development refers here to institutional arrangements that bring cross-sector effects into the rules governing policy appraisal, authority, financing, measurement, accountability, and learning (5–36). Sustainable development defines the normative objective; integrated governance concerns how institutions pursue that objective when policy choices have consequences across sectors, populations, places, and time horizons.

Coordination alone is insufficient. It can bring institutions together while leaving mandates, incentives, budget rules, evidence systems, and lines of accountability unchanged. Integrated governance requires cross-sector effects to influence decisions: who has authority to resolve competing objectives, how shared benefits are financed, how spillovers and distributional consequences are measured, who is accountable for harms transferred across domains, and how implementation changes when evidence identifies unintended effects (14–18, 34–36).

The concept builds on established traditions. Policy coherence for sustainable development addresses alignment across sectors, levels of governance, borders, and time horizons (15–18). Health in All Policies brings health and equity considerations into decisions beyond the health sector (19–24). One Health links human, animal, and ecosystem health and exposes the institutional difficulty of governing across disciplinary and administrative boundaries (25–27). Systems thinking and implementation science emphasize feedback, context, non-linearity, and adaptation (29, 32, 33), whereas health-system strengthening contributes lessons on resilience, accountability, and learning (30, 31).

The proposed framework integrates these institutional lessons around the problem of SDG interdependence. Its six functions specify the capacities required to connect decision authority, resources, evidence, accountability, and learning across policy domains.

### 4.3 Why interdependence requires institutional design

The 2030 Agenda treated integration as foundational, yet many implementation systems continued to operate through sectoral mandates (1, 5, 12). This disjunction matters because interactions among goals can generate co-benefits as well as trade-offs. Progress in one domain may accelerate progress elsewhere, but it may also transfer costs to other sectors, populations, countries, or future generations (5–11, 14–18).

SDG interlinkage research shows that the goals operate as a network of targets and interactions rather than as isolated commitments (5–11). Network analysis has demonstrated the high connectivity of SDG targets, while subsequent work has emphasized the need to anticipate synergies and trade-offs before policies are adopted (5, 6, 11). Empirical studies further show that these interactions vary by context, income level, and indicator choice, requiring decision systems that interpret interdependence in relation to place, capacity, and policy design (7, 8).

Systems thinking and governance theory reinforce this institutional argument. Development outcomes emerge from relationships, feedback loops, and delayed effects, while complex collective-action problems often require distributed authority, collaboration, and learning across levels of government (29, 34–36). Institutions therefore need to assess how policies alter risk, opportunity, resilience, and distribution across the wider system alongside their contribution to individual targets (29, 34–36).

Policy coherence for sustainable development provides one route for this shift. OECD guidance calls for strategic vision, whole-of-government coordination, policy integration, stakeholder engagement, subnational alignment, and monitoring systems that account for domestic, transboundary, and long-term effects (16, 17). Recent assessments show that governments continue to struggle with policy divergences, spillovers, and transboundary effects (18). The central challenge now is building institutions with the capacity to govern the consequences of SDG interdependence across policy domains (15–18).

## 5 Public health and the governance of interdependence

Public health connects upstream policy choices with population-level outcomes and their distribution. Health is produced through exposures, living conditions, services, rights, markets, behaviors, and institutions (19–24, 37). Ministries of health can strengthen services, but cannot alone secure clean air, safe housing, healthy food environments, decent work, healthy mobility, or protection from climate-related shocks (19, 37–40). Public health consequently makes visible the effects of decisions that are administratively dispersed but biologically and socially cumulative.

Progress on SDG 3 illustrates this dependence because maternal and child health, non-communicable diseases, mental health, injury prevention, infectious-disease control, universal health coverage, and health security are shaped by decisions across sectors (4, 19–24). Climate policy affects heat exposure, air pollution, food security, infectious-disease risk, and health-system resilience (38). Urban and transport policy shape physical activity, road injury, air quality, access, and social inclusion (39). Food policy affects diets, chronic disease, affordability, livelihoods, land use, and environmental sustainability (40). Social protection can reduce poverty and vulnerability while influencing health-service use and health outcomes in low- and middle-income countries (41).

Public health also offers practical traditions for governing interdependence. Health in All Policies provides a model for embedding health considerations in public policies across sectors, seeking synergies, and avoiding harmful health impacts (19). WHO guidance on multisectoral collaboration identifies governance, accountability, leadership, working practices, and resources as conditions for sustained intersectoral action (20). Earlier WHO work described cabinet committees, interdepartmental units, joint budgeting, and delegated financing as mechanisms for embedding health within government decision-making (21).

Peer-reviewed evidence reinforces these institutional lessons. Analyses of Health in All Policies show that governance structures, partnerships, community engagement, monitoring arrangements, political leadership, institutional mandates, accountability mechanisms, and financing shape whether cross-sector commitments become practice (22–24). One Health adds a complementary model by linking human, animal, and ecosystem health, while also showing how sectoral, professional, and institutional silos, financing gaps, and power asymmetries can limit integrated action (25–27). These limits are instructive: integration produces public value only when it is supported by ethical accountability, fair access to data, transparent priority setting, and safeguards for communities that bear disproportionate risk (27).

Health-system strengthening and implementation science add a further layer of policy learning. Universal health coverage requires systems capable of improving quality, equity, efficiency, accountability, resilience, and sustainability (30). Integrated health-system strengthening emphasizes locally integrated delivery, data systems, impact evaluation, and feedback loops that support adaptation (31). Implementation science similarly foregrounds context, implementation processes, feedback, and adaptation when evidence is translated into practice (32, 33). For the agenda beyond 2030, the issue is therefore broader than whether health systems can improve SDG 3 outcomes. It is whether institutions can govern the wider determinants on which those outcomes depend (19–24, 30–33).

These institutional lessons are not confined to SDG 3 or to ministries of health. Similar requirements arise in climate adaptation, food systems, urban development, social protection, and other domains in which outcomes depend on decisions distributed across sectors and levels of governance. The analysis centers on public health. Application of the six functions to other domains requires contextual and empirical assessment.

## 6 Policy options and a framework for integrated governance

### 6.1 Policy options and implications for public health governance beyond 2030

The policy question is how recognized interdependence can be incorporated into institutional practice. Three broad options can be understood as a staged continuum: interlinkage reporting strengthens the evidence base; policy-coherence and multisectoral mechanisms bring cross-sector effects into decision processes; and explicit governance functions embed them in mandates, budgets, measurement systems, accountability arrangements, and implementation (12–18).

### 6.2 Option 1: retain sectoral SDG accountability with incremental interlinkage reporting

The first option retains the current sectoral architecture of goals, targets, and indicators while improving interlinkage mapping and narrative reporting. This approach is administratively feasible because it builds on existing SDG reporting systems, statistical capacities, and voluntary national review processes (1, 5–11).

Its limitation is institutional. Interlinkage reporting can describe how goals interact, but it does not create authority to arbitrate trade-offs, align budgets, or hold institutions accountable for harms transferred across sectors, populations, borders, or time horizons (14, 16–18). For public health, this option risks retaining SDG 3 as a health-sector performance measure and failing to use health outcomes to assess whether development institutions are addressing their wider determinants (4, 19–24).

### 6.3 Option 2: strengthen policy coherence and multisectoral governance

The second option strengthens policy coherence for sustainable development, Health in All Policies, and whole-of-government coordination. It embeds health, equity, environmental, and long-term considerations into non-health policy processes through mechanisms such as cabinet committees, interministerial units, integrated policy appraisal, joint budgeting, shared indicators, and subnational coordination (16–24, 28).

This option moves integration from reporting toward decision-making. Its effectiveness depends on authority, budgetary leverage, and accountability. Without these conditions, coherence mechanisms can remain advisory; committees may convene actors without changing incentives; impact assessments may occur too late in the policy process; and shared indicators may be reported without consequences for institutions that externalize costs to other sectors or populations (18, 22–24).

### 6.4 Option 3: institutionalize integrated governance through explicit functions

The third option institutionalizes integrated governance through explicit functions. It retains sustainable development as the normative objective while specifying the institutional capacities required to act on interdependence. It brings together policy coherence for sustainable development, Health in All Policies, One Health, health-system strengthening, implementation science, and governance theory within an operating framework for the agenda beyond 2030 (15–36).

This option is the most institutionally demanding. Governments and international institutions would need to specify how cross-sector effects enter policy appraisal, where authority for resolving trade-offs resides, how budgets recognize shared outcomes and prevention, how spillovers and distributional consequences are measured, who is accountable for regressive outcomes, and how evidence triggers policy revision. Adoption would necessarily be staged and adapted to political authority, administrative capacity, fiscal space, data infrastructure, and levels of decentralization. Table 1 summarizes the implications of the three options.

**Table 1:** Policy options for governing SDG interdependence beyond 2030: implications for public health.

Policy option	Core logic	Main strengths	Main limitations for public health equity	Best use beyond 2030
Option 1: incremental interlinkage reporting	Retain the sectoral SDG architecture while improving reporting on selected synergies, trade-offs, and spillovers.	Feasible and builds on existing SDG reporting, dashboards, statistical systems, and voluntary national review processes.	Describes interdependence without necessarily changing authority, budgets, incentives, or accountability.	A minimum improvement to the evidence base, but insufficient as the main reform pathway.
Option 2: stronger policy coherence and multisectoral governance	Embed health, equity, environmental, and long-term considerations into cross-sector policy processes.	Moves integration closer to decision-making and aligns with PCSD, Health in All Policies, and multisectoral action traditions.	May remain advisory or procedural without budgetary leverage, arbitration authority, and accountability for cross-sector effects.	A feasible transitional pathway where whole-of-government mechanisms already exist or can be strengthened.
Option 3: integrated governance through explicit institutional functions	Institutionalize coherence, arbitration, shared-outcome financing, interlinkage-aware measurement, equity-sensitive accountability, and adaptive learning.	Translates recognized interdependence into an operating framework that connects decision authority, resources, evidence, accountability, and learning.	Requires political authority, administrative capacity, fiscal and data infrastructure, and adaptation to national and subnational contexts.	A long-term reform direction implemented through staged institutional change.

### 6.5 A six-function framework for integrated governance beyond 2030

The framework translates recognized interdependence into six institutional functions that can be embedded in policy appraisal, decision authority, budgets, measurement systems, accountability arrangements, and implementation review (1, 10, 15–18, 32–36). The functions were identified from recurrent implementation requirements across the reviewed literatures: aligning objectives, resolving trade-offs, allocating resources, measuring cross-sector effects, accounting for distributional consequences, and using feedback for course correction (5–18, 21–24, 29–36, 44).

The contribution lies in their functional integration. Interdependence, policy coherence, multisectoral action, and adaptive governance are established concepts; the framework specifies how they relate to a set of institutional tasks that must be performed when interactions among policy domains become consequential for population health and equity. Coherence and arbitration concern decision-making; shared-outcome financing and interlinkage-aware measurement connect resources and evidence; and equity-sensitive accountability and adaptive learning connect institutional responsibility with policy revision. Table 2 summarizes these functions and their implications for public health.

**Table 2:** Six functions of integrated governance for sustainable development: implications for public health.

Area of institutional practice	Function	Sectoral SDG logic	Integrated governance requirement	Public health and equity implication
Decision-making	Coherence	Ministries and agencies align activities around separate targets.	Policy processes assess cross-sector effects before and during implementation.	Health, equity, and sustainability are built into decisions beyond the health sector.
Decision-making	Arbitration	Trade-offs are managed informally or after problems emerge.	Mandated bodies resolve conflicts among economic, social, environmental, and health objectives using explicit criteria and evidence.	Public health evidence informs decisions when policy objectives compete.
Resources and evidence	Shared-outcome financing	Budgets reward sectoral outputs.	Budget and investment rules recognize co-benefits, prevention, and long-term public value.	Investments outside health are valued for health and equity gains.
Resources and evidence	Interlinkage-aware measurement	Dashboards report progress goal by goal.	Measurement systems assess synergies, trade-offs, spillovers, and distributional effects.	Evidence identifies who benefits, who bears costs, and who remains excluded.
Accountability and learning	Equity-sensitive accountability	Reporting focuses on aggregate progress.	Institutions are answerable for cross-sector harms, exclusion, and regressive effects.	Disaggregated data and participation make inequities visible and actionable.
Accountability and learning	Adaptive learning	Targets are reviewed periodically.	Feedback loops trigger policy adjustment when harms, exclusions, or unintended effects emerge.	Institutions revise policies when evidence shows harm, exclusion, or unintended effects.

#### 6.5.1 Coherence

Coherence gives institutional form to integration. Coordination may convene institutions, but coherence changes how evidence, incentives, and authority shape decisions. In public health policy, this means that major decisions outside the health sector are assessed for their health, equity, environmental, fiscal, and long-term implications before adoption and during implementation (16–18, 21–24).

#### 6.5.2 Arbitration

Alignment is insufficient when development objectives conflict. Choices about growth, fiscal constraint, environmental protection, social protection, and health equity often involve real trade-offs. Whole-of-government bodies need authority to resolve these conflicts using explicit criteria and evidence. Public health evidence should be visible when governments weigh economic development against air pollution, unsafe housing, unhealthy food environments, climate-related risk, or diminished access to services (14, 18, 37–41).

#### 6.5.3 Shared-outcome financing

Shared-outcome financing aligns budgets and investment decisions with co-benefits, prevention, and long-term public value across sectors. Many health gains originate outside the health sector, including through transport, housing, education, food systems, social protection, and climate adaptation. Budget rules, investment cases, and performance agreements should therefore recognize the health and equity value of policies delivered by non-health sectors (19, 24, 30, 38–41).

#### 6.5.4 Interlinkage-aware measurement

Interlinkage-aware measurement supports decision-making and learning. A complete map of all SDG interactions is rarely available before action is needed. Decision makers require context-specific tools that identify relevant synergies, trade-offs, spillovers, and distributional effects. Public health can contribute methods from health impact assessment, burden-of-disease analysis, health equity assessment, implementation science, and health-system performance monitoring (8, 11, 21, 31–33).

#### 6.5.5 Equity-sensitive accountability

Equity-sensitive accountability anchors integration in distributional consequences. Integrated governance arrangements can reproduce power asymmetries when actors with greater technical capacity, data control, or political influence dominate decision processes (18, 27). Credible accountability requires meaningful participation, transparent priority setting, disaggregated data, and protection from regressive effects. These safeguards help prevent integrated governance from becoming technocratic alignment that overlooks lived risk and unequal exposure to harm (23, 24, 37, 41).

#### 6.5.6 Adaptive learning

Adaptive learning links evidence to course correction. Complex systems rarely respond to interventions in linear ways (29, 33). Institutions beyond 2030 will need to monitor implementation, identify unintended effects, revise policies when evidence shows harm or exclusion, and communicate learning across sectors and levels of governance (32, 33).

## 7 From interlinkage reporting to decision-oriented accountability

Measurement will be central to the development agenda beyond 2030. The SDG indicator framework created a shared statistical infrastructure, but sectoral dashboards can obscure the interactions through which progress in one area affects risk, equity, and outcomes elsewhere (1, 5–8). Reviews of SDG interlinkages show recurring patterns of synergies and trade-offs across goals, including interactions at target and indicator levels (8). Accountability beyond 2030 therefore requires evidence systems that identify interdependence and connect the findings to policy appraisal, authorization, financing, accountability, and implementation (8, 16–18).

Such evidence systems need to capture effects beyond the sector, place, or time horizon in which a policy is adopted. A policy may appear successful domestically or in the short term while shifting environmental, fiscal, or health burdens to other populations, future generations, or weaker institutions (16–18). Measurement should therefore support institutional learning: identifying effects, revising assumptions, and enabling course correction when policies produce harm or exclusion (16–18, 32, 33). Interlinkage-aware measurement need not map every possible interaction before action is taken; its purpose is to identify effects material to the decision, make their distribution visible, and connect evidence to institutional responsibility and course correction.

Public health has long confronted this measurement problem. Vertical programs may generate measurable outputs without demonstrating population-level impact when data systems, feedback loops, and equity analysis remain fragmented (31). A vaccination campaign, urban transport reform, food tax, climate adaptation plan, or social protection policy may affect health through multiple pathways. A narrow indicator may record one output while missing effects on access, trust, risk exposure, gender equity, fiscal protection, or future vulnerability (19, 31, 38–41).

Equity makes this challenge more demanding. The pledge to leave no one behind requires disaggregated data (1), yet many systems still lack sufficient disaggregation by income, gender, age, geography, disability, migration status, and other axes of vulnerability. The Lancet Commission on peaceful societies through health equity and gender equality emphasized the need for feedback loops and documented persistent data limitations for equity-sensitive accountability (44). Integrated governance therefore requires measurement systems able to show who benefits, who bears costs, what trade-offs emerge, and how institutions respond when evidence reveals harm or exclusion (1, 37, 44).

The Pact for the Future’s commitment to measures of progress beyond gross domestic product creates an opportunity to redesign measurement around public value rather than economic output alone (3). From a public health perspective, such measures should guide decisions. A parallel reporting exercise would add little value. They should help institutions connect health, equity, sustainability, resilience, and performance in ways that shape policy choices (3, 16–18, 37, 44).

## 8 Application, implementation, and boundaries of the framework

### 8.1 Climate adaptation and health

Climate adaptation illustrates how the six functions can translate recognized interdependence into institutional practice. Coherence would bring health, urban planning, housing, water, labor, social-protection, and finance authorities into a shared assessment of climate-related health risks. Arbitration would make trade-offs explicit when infrastructure, economic development, environmental protection, and equity objectives conflict. Shared-outcome financing would support investments such as heat-resilient housing, urban cooling, early-warning systems, and protection for outdoor workers, for which benefits accrue across sectors. Interlinkage-aware measurement would assess heat exposure, air quality, service disruption, vulnerability, and distributional effects. Equity-sensitive accountability would identify populations that remain most exposed and least protected, whereas adaptive learning would allow plans to be revised as hazards, health risks, and evidence evolve (16–18, 21–24, 32, 33, 38, 41, 44).

### 8.2 Staged implementation

Implementation does not require immediate reform of the entire SDG or governmental architecture. Initial application should focus on bounded policy areas in which interdependence is already empirically and politically visible, including climate adaptation, food systems, urban development, social protection, and health financing (38–41).

A staged approach would define the policy problem, identify the sectors and populations affected, and assess which of the six functions are absent or weak. Institutional responses could then be developed around specific decisions, with coordination tailored to the policy problem. Depending on context, the entry point might be cabinet-level policy appraisal, joint budgeting, subnational planning, health or equity impact assessment, or strengthened accountability for cross-sector effects (16–24).

This approach reduces the risk that integration becomes an additional layer of committees and reporting without corresponding authority, resources, or accountability. Early applications should be used to assess feasibility, document institutional behavior, identify unintended effects, and refine the framework before wider adoption.

### 8.3 Boundaries and contextual adaptation

The framework supports policy analysis and institutional design. It does not serve as a universal indicator set, validated instrument, or prescriptive organizational model. Interlinkages are context dependent, data systems remain uneven, and many governments have limited capacity to trace causal pathways across sectors, borders, and time horizons (7, 8, 18). The objective is to make material relationships sufficiently visible to inform decisions, monitor effects, and revise policies when evidence indicates harm, exclusion, or weak effectiveness.

The six functions may be institutionalized differently according to constitutional arrangements, political authority, administrative capacity, fiscal space, data infrastructure, and levels of decentralization. Their relevance beyond public health requires empirical evaluation. Comparative research is needed to determine how the functions operate in different settings, which combinations are feasible, and whether stronger integrated-governance capacity is associated with more equitable and sustainable outcomes.

These limits are integral to the interpretation of the framework. It specifies institutional functions that require attention under conditions of interdependence; it does not prescribe a single model of governance.

## 9 Actionable recommendations

The six functions imply corresponding institutional actions. The recommendations below specify functions rather than mandatory organizational forms, allowing adaptation to different constitutional, fiscal, political, and administrative settings.

### 9.1 Require integrated appraisal for major policy decisions

National governments, central planning agencies, and ministries of finance should require major policies, investment programs, and legislative proposals to assess health, equity, environmental, fiscal, intergenerational, and transboundary effects before approval and during implementation. Appraisal should shape policy design, budget negotiation, and monitoring, rather than function as a late-stage compliance requirement (16–18, 21–24).

### 9.2 Establish arbitration mechanisms for SDG trade-offs

Cabinets, planning councils, or equivalent whole-of-government bodies should establish procedures for resolving conflicts among economic, social, environmental, and health objectives. Arbitration should be guided by explicit criteria, public health evidence, equity analysis, and transparent documentation of who benefits, who bears costs, and how harms will be mitigated (14, 18, 34–37).

### 9.3 Align financing with shared outcomes and prevention

Ministries of finance, development partners, and sectoral agencies should design budget rules, investment cases, and performance agreements that recognize co-benefits, prevention, and long-term public value. Financing arrangements should enable non-health sectors to invest in policies that generate health and equity gains, including housing, transport, food systems, education, climate adaptation, and social protection (19, 24, 30, 38–41).

### 9.4 Build measurement systems that capture interdependence

National statistical offices, health ministries, planning agencies, and global monitoring bodies should move beyond goal-by-goal dashboards toward decision-oriented evidence on synergies, trade-offs, spillovers, and distributional effects. Disaggregated data should be linked to policy appraisal, budget decisions, and accountability mechanisms, rather than treated only as reporting outputs (8, 11, 16–18, 44). Measurement should be proportionate and decision specific; not every interaction can or should be quantified before action.

### 9.5 Institutionalize equity-sensitive accountability

Governments, public health institutions, and civil society actors should ensure meaningful participation, transparent priority setting, and mechanisms for identifying and responding to regressive effects. Communities exposed to cumulative disadvantage should have a role in defining risks, interpreting evidence, and holding institutions accountable for harms created across sectors (23, 24, 27, 37, 44). Accountability arrangements should specify how evidence of harm or exclusion leads to an institutional response rather than ending with disclosure.

### 9.6 Embed adaptive learning in implementation

Implementing institutions should monitor unintended effects, revise policies when evidence shows harm or exclusion, and communicate learning across sectors and levels of governance. Adaptive learning should be treated as a core governance function, not as a post-hoc evaluation activity (29, 32, 33). Review triggers, decision rights, and responsibility for policy revision should be defined prospectively rather than left to discretionary post-hoc evaluation.

## 10 Discussion

This review addresses an institutional gap in the implementation of sustainable development. The interconnectedness of the SDGs is established; the contribution of the framework is to specify the functions through which that interdependence can influence policy decisions, resource allocation, accountability, and implementation (1, 5–18). Coherence, arbitration, shared-outcome financing, interlinkage-aware measurement, equity-sensitive accountability, and adaptive learning together provide a functional account of the capacities required to govern interactions among policy domains.

The framework integrates insights from bodies of scholarship that are often considered separately. SDG-interlinkage research identifies synergies, trade-offs, and spillovers; policy coherence for sustainable development addresses alignment across sectors, levels of governance, borders, and time horizons; Health in All Policies and One Health provide experience in intersectoral governance; implementation science and systems thinking address context, feedback, and adaptation; and governance theory addresses authority, collective action, and institutional design (5–36). The framework connects the institutional implications of these traditions within a common operating logic.

Public health is particularly informative because health outcomes accumulate the consequences of decisions made across sectors, whereas health inequities reveal how risks, protections, and opportunities are distributed (4, 19–24, 37–41). The same institutional problem, however, extends beyond health. Climate adaptation, food systems, urban development, and social protection similarly depend on coordinated decisions across sectors and levels of government. The framework’s applicability to these domains requires contextual assessment.

The framework also clarifies the limits of measurement. Indicators remain indispensable. On their own, they cannot arbitrate trade-offs, align budgets, assign responsibility, or trigger course correction. Evidence becomes institutionally consequential only when it enters decision processes, shapes resource allocation, establishes accountability, and leads to policy revision when harm or exclusion becomes visible (8, 16–18, 32, 33, 44).

Equity is central to this institutional use of evidence. Integrated governance can reproduce or reinforce power asymmetries if participation is symbolic, data are controlled by more powerful institutions, or aggregate progress obscures unequal exposure to harm (18, 23, 24, 27, 37, 41). Equity-sensitive accountability is a condition of legitimate integrated governance.

The framework is a policy synthesis and design heuristic that requires empirical testing and contextual refinement. Future research should compare how countries institutionalize coherence, arbitration, financing, measurement, accountability, and adaptive learning; how these functions vary across governance and resource settings; and whether stronger configurations are associated with more equitable progress on SDG 3 and related goals (8, 11, 16–18, 29, 32–36).

## 11 Conclusion

The agenda beyond 2030 must equip institutions to deliver sustainable development under conditions of interdependence. Public health makes the consequences of fragmented governance visible because health and equity are shaped by interacting decisions across social, economic, environmental, fiscal, and service systems, while responsibility for those decisions remains distributed among institutions.

A credible future agenda should therefore complement goals and indicators with explicit governance functions. Coherence, arbitration, shared-outcome financing, interlinkage-aware measurement, equity-sensitive accountability, and adaptive learning specify the capacities required when policy choices generate cross-sector effects, trade-offs, spillovers, and unequal exposure to harm. The transition beyond 2030 provides a policy window to move from recognition of interdependence to institutional action. Its credibility will depend on the goals retained or adopted and on whether authority, resources, evidence, accountability, and learning are organized to protect health and equity across interconnected systems.
